# Auditory processing in dysphonic children

**DOI:** 10.1590/S1808-86942011000300015

**Published:** 2015-10-19

**Authors:** Mirian Aratangy Arnaut, Caroline Vieira Agostinho, Liliane Desgualdo Pereira, Luc Louis Maurice Weckx, Clara Regina Brandão de Ávila

**Affiliations:** 1 MSC in Sciences - Graduate Program in Human Communication Disorders - Speech and Hearing Department - University of São Paulo; Audiologist and Speech Therapist; 2 MSC in Sciences - Graduate Program in Human Communication Disorders - Speech and Hearing Department - University of São Paulo; Audiologist and Speech Therapist; 3 Associate Professor - Program in Human Communication Disorders - Speech and Hearing Department - University of São Paulo; Professor of Hearing Disorders - Department of Speech and Hearing Therapy - UNIFESP; 4 Full Professor of Otorhinolaryngology - Federal University of São Paulo; 5 PhD in Human Communication Disorders - Federal University of São Paulo (UNIFESP); Associate Professor at the Department of Speech and Hearing Therapy - UNIFESP

**Keywords:** evaluation, child, dysphonia, auditory perception

## Abstract

Contemporary cross-sectional cohort study. There is evidence of the auditory perception influence on the development of oral and written language, as well as on the self-perception of vocal conditions. The auditory system maturation can impact on this process.

**Objective:**

To characterize the auditory skills of temporal ordering and localization in dysphonic children.

**Materials and Methods:**

We assessed 42 children (4 to 8 years). Study group: 31 dysphonic children; Comparison group: 11 children without vocal change complaints. They all had normal auditory thresholds and also normal cochleo-eyelid reflexes. They were submitted to a Simplified assessment of the auditory process (Pereira, 1993). In order to compare the groups, we used the Mann-Whitney and Kruskal-Wallis statistical tests. Level of significance: 0.05 (5%).

**Results:**

Upon simplified assessment, 100% of the Control Group and 61.29% of the Study Group had normal results. The groups were similar in the localization and verbal sequential memory tests. The nonverbal sequential memory showed worse results on dysphonic children. In this group, the performance was worse among the four to six years.

**Conclusion:**

The dysphonic children showed changes on the localization or temporal ordering skills, the skill of non-verbal temporal ordering differentiated the dysphonic group. In this group, the Sound Location improved with age.

## INTRODUCTION

It is not unusual for dysphonic children to be unable to judge the characteristics of their own voices; therefore, they depend on external signs, such as negative reactions from listeners so as to perceive aspects associated with their voice production and quality. Peripheral hearing losses associated to voice changes, alter one's voice perception^1^. To identify the dysphonic patient's perceptive-auditory capacity conditions may be very valuable to establish treatment goals in vocal rehabilitation. Moreover, these perception skills are important for self-monitoring of the new vocal conditions, even if subconsciously.

In fact, some studies have shown the existence of a relationship between auditory perception changes and dysphonia. The performance in auditory processing assessment in of dysphonic children, compared to that of other children without hearing and speech complaints, showed a statistically significant difference between the groups^2^, ^3^, ^4^, pointing out that vocal rehabilitation may be subject to the auditory perception of one's own voice.

Auditory perception assessment is carried out by a set of special test^1^, ^5^, ^6^, ^7^ and it is incorporated to the diagnosis of most of the human communication disorders^1^. Some studies discuss the need to take into account issues associated with the auditory system maturation in the assessment of development disorders^7^, ^8^, ^9^, because these children may also present delays in the development of their auditory skills^10^, ^11^. Today, the importance of temporal processing is recognized, for example in speech perception^12^. The Simplified Assessment of the auditory function proposal has proven capable of characterizing groups of children with learning disorders^9^, ^11^, ^13^, ^14^, ^15^. Easy to apply, it could complement the tasks which usually assess dysphonic children's voices and bring about important information about their sound source localization auditory skills and sound pattern auditory memory. The latter would influence the learning of language sound codes, the verbal sound pattern memory involves not only the retention of immediately perceived words, enabling the transmission of ideas, but it also enables the learning of new words. The auditory memory for non-verbal sounds has the basic function of distinguishing sound relations such as prosody, volume, intensity, voice tone, and synthetizing it in melodic structures^16^. All these skills are implicated in vocal auditory perception.

The goal of the present study was to characterize auditory skills associated with the localization and recognition of sound stimuli sequences in dysphonic children in search of evidence of the influence of auditory perception on vocal quality, taking into account the age range and the auditory system maturation.

## MATERIALS AND METHODS

This paper was approved by the Ethics in Research Committee, under protocol # 019/03, which was started after the participants' parents or guardians signed the Informed consent form.

We initially assessed 56 children, boys and girls with vocal complaints. At the time of the assessments, their ages varied between 4 and 8 years. The sample was collected during the period of one year.

We established, beforehand, the following inclusion criteria: otorhinolaryngology diagnosis of dysphonia; normal auditory thresholds; no complaints or hints of changes associated with speech development, language and hearing; no complaints or hints of neurologic changes or sensory-motor cognitive deficits.

The children were assessed by an ENT physician for the clinical diagnosis of vocal complaints. On the same date, they went through speech assessment and Simplified Assessment of the Auditory Processing (SAAP). Audiometric assessments were carried out by appointment, two weeks after the initial visit. 19 children were taken off this sample because they had speech changes or because they did not come for their audiological assessment. The Study Group (SG) was initially made up of 37 dysphonic children (22 boys).

Another group, called Comparison Group (CG) was made up by 11 children (two boys), of the same age range as those from the SG, recruited from private schools in the city of São Paulo. None of them complained of vocal change onsets and met the other criteria established for sample inclusion. The lack of complaints or vocal changes was confirmed by voice auditory perception carried out by the researcher during initial contact with the child. The participants volunteered to take part in the study, and they did, after their parents signed the Informed Consent Form.

Given the number difference between the groups, they were comparable as to gender (Fisher's Exact Test, *p*=0.058) and age (Mann-Whitney test, p= 0.510) and they were similar as to these variables.

### Procedures

In a silent room, the SG and CG participants were individually submitted to the Simplified Auditory Processing Assessment, proposed by Pereira (1993): Sound Source Localization (SSL); Non-Verbal Sound Sequence Memory (NVSSM); Verbal Sounds Sequence Memory (VSSM); and Cochlear-Blink Reflex (CBR).

We used the stimulus of a single high intensity bang (around 90 dB) on the agogo, to investigate CBR. Of the 37 children assessed in this test, 6 (5 boys) were taken off the sample for not having the cochlear-blink reflex. The final SG was made up of 31 dysphonic children (17 boys).

In the Sound Localization test, we used the sound of a rattle as sound stimulus presented in five directions: right side, above, behind, left side and in front of the participant, who kept his eyes shut during the assessment. Each correct answer was equivalent to 20% of the likelihood of responses.

In the NVSSM test, we used up to four sound instruments, of broad frequency spectrum, being: bell, agogo, rattle and coco. For the children up to 06 years of age we executed sequences of three stimuli, and for children of 7 and 8 years, sequences of four. The sounds of these instruments were presented to each participant in three different sequences, produced with presentation order inversion.

In the VSSM test, the PA - TA - CA syllables were presented to the child in three different sequence orders, modified only by the presentation order inversion. We used the same scoring criteria used in the NVSSM test. Both in the VVSM as well as in the NVSSM tasks, each identified sequence was equal to 33.33% of the correct answers, and 100% corresponded to getting the three sequences right; 66.66% for two; 33.33% when only one sequence was right and 0% when the child was not successful in pointing to the sequence of instruments heard.

The normality reference criterion used for the verbal and non-verbal sound sequence tasks, regardless of the number of sound stimuli, was of at least two correct sequences in three attempts. And, for the Sound Localization Test, we used the sound source identification tasks on the right and on the left, and at least two of the three sound sources localized above, in front of and behind the child.

All the responses observed for the above-mentioned tasks were written down in an appropriate protocol, which had the other information from each participant (age, gender, ENT and speech and hearing diagnoses; auditory thresholds from both ears). The correct answers were computed in percentage values for later comparison between the groups.

For the statistical calculation we considered the percentage of correct answers in each one of the tests done. In order to do the statistical comparison between the groups we employed the following tests: Fisher' Exact, Mann-Whitney and Kruskal-Wallis, and the Bonferroni's rule. We used the SPSS (Statistical Package for Social Sciences) software, in its 13.0 version. The level of significance was established in 0.05 (5%) for the three tests. We used the Bonferroni's rule to correct the significance level which was altered in function of the number of possible combinations. Thus, since we have ten age two-by-two combinations, the 0.05 value was divided by 10, resulting in another value less than or equal to 0.005. The statistically significant values were marked by an asterisk (*).

## RESULTS

Upon the distribution and description of the children according to age, gender, ENT and speech and hearing diagnoses and the results from the Auditory Processing Assessment, it is possible to notice that of the children from the SG, 74.2% had organic-functional dysphonia, while only 25.8% had functional dysphonia. As to Auditory Processing, 100% of the children from the CG had normal results; while in the SG, 61.29% had a normal result.

On Table 1 we can notice the distribution and description of the children from the SG and the CG according to age, gender, ENT and speech and hearing diagnoses and the results from the Auditory Processing Assessment. We notice that most of the children (74.2%) had organic-functional dysphonia and the others had functional dysphonia. As far as Auditory Processing goes, 100% of the children from the CG had normal results, while in the SG, 61.29% had normal results.

**Table 1:** SG and CG distribution by gender, age, speech and hearing diagnosis and performance in the Simplified Auditory Processing Assessment.

	AGE	GENDER	DIAGNOSIS	NVSSM	VSSM	SLT	CBR	SAPA result
SG	4	F	organic-funnctional	0%	66.66%	100%	100%	altered
	4	M	organic-funnctional	33.33%	100%	80%	100%	altered
	4	F	organic-funnctional	33.33%	0%	80%	100%	altered
	5	F	organic-funnctional	0%	66.66%	100%	100%	altered
	5	M	organic-funnctional	66.66%	0%	80%	100%	altered
	5	M	organic-funnctional	66.66%	100%	100%	100%	normal
	5	M	organic-funnctional	66.66%	100%	60%	100%	altered
	5	M	organic-funnctional	100%	0%	100%	100%	altered
	6	M	organic-funnctional	66.66%	100%	100%	100%	normal
	6	F	functional	66.66%	66.66%	100%	100%	normal
	6	F	functional	33.33%	100%	100%	100%	altered
	6	M	organic-funnctional	66.66%	100%	80%	100%	normal
	6	F	functional	100%	100%	100%	100%	normal
	6	F	organic-funnctional	100%	66.66%	100%	100%	normal
	6	F	organic-funnctional	100%	100%	80%	100%	normal
	6	F	funcional	100%	66.66%	100%	100%	normal
	6	M	organic-funnctional	100%	66.66%	80%	100%	normal
	6	M	organic-funnctional	66.66%	100%	100%	100%	normal
	6	M	organic-funnctional	0%	66.66%	80%	100%	altered
	7	F	functional	100%	100%	100%	100%	normal
	7	M	organic-funnctional	100%	100%	100%	100%	normal
	7	M	organic-funnctional	66.66%	100%	100%	100%	normal
	7	F	organic-funnctional	66.66%	66.66%	100%	100%	normal
	7	M	organic-funnctional	100%	66.66%	100%	100%	normal
	7	M	organic-funnctional	33.33%	100%	60%	100%	altered
	7	M	organic-funnctional	100%	100%	100%	100%	normal
	8	F	functional	100%	100%	60%	100%	normal
	8	M	functional	100%	66.66%	100%	100%	normal
	8	M	organic-funnctional	100%	100%	80%	100%	normal
	8	F	functional	100%	33.33%	100%	100%	altered
	8	F	organic-funnctional	100%	100%	80%	100%	normal
CG	4	F	-	100%	100%	80%	100%	normal
	5	M	-	100%	100%	100%	100%	normal
	5	F	-	66.66%	66.66%	100%	100%	normal
	6	M	-	100%	66.66%	100%	100%	normal
	6	F	-	100%	100%	100%	100%	normal
	6	F	-	100%	100%	100%	100%	normal
	7	F	-	100%	66.66%	100%	100%	normal
	7	F	-	100%	66.66%	100%	100%	normal
	7	F	-	66.66%	100%	100%	100%	normal
	8	F	-	100%	100%	100%	100%	normal
	8	F	-	100%	66.66%	100%	100%	normal

Legend: SG= Study Group; CG= control group; FONO= speech and hearing diagnosis; NVSM= non-verbal sequential memory; VSM=verbal sequential memory; SLT= sound localization test; CBR= cochlear-blink reflex; M = male; F=female SAPA= Simplified Auditory Processing Assessment

Table 2 shows the descriptive measures and comparisons of the percentage mean values of correct answers obtained from the Simplified Auditory Processing Assessment tests. It is noticed that the statistically significant difference in the NVSSM test, dysphonic children had the worst performance.

**Table 2:** Distribution of the correct answers percentage mean value in the Simplified Auditory Processing Assessment tests seen in the SG and CG children.

Prova	Group	N	Mean	Standard deviation	Minimum	Maximum	Percentile 25	Median	Percentile 75	*p*-value
NVSM	ST	31	72.04%	33.44%	0.00%	100%	66.66%	66.66%	100%	
	CG	11	93.94%	13.49%	66.66%	100%	100%	100%	100%	0,040*
VSM	ST	31	77.42%	31.49%	0.00%	100%	66.66%	100%	100%	
	CG	11	84.85%	17.41%	66.66%	100%	66.66%	100%	100%	0,760
SLT	ST	31	90.32%	13.54%	60.00%	100%	80%	100%	100%	
	CG	11	98.18%	6.03%	80.00%	100%	100%	100%	100%	0,067

Legend: NVSM=Non-verbal sequential memory; VSM=verbal sequential memory; SLT= sound localization test; M= male; F=female; Kruskall-Wallis test;

* statistically significant p-value fixed on 0.05

The intragroup comparison of the different tests done, according to age, showed in the SG the presence of a statistically significant difference among the ages in the NVSSM test analysis, and the worst performance was seen among 4 year-olds. In the CG, it was possible to notice a worse performance among 4 year olds in the Sound Source Localization task.

Table 3 shows the intragroup performance of the SG and CG children in the different tests carried out, according to age. We noticed in the SG the presence of statistically significant difference among the ages in the NVSSM test, and the worst performance was noticed among 4 year-olds. In the CG we noticed a worse performance among 4 year-olds in the Sound Source Localization task.

**Table 3:** Descriptive values of the correct answers from the Simplified Auditory Processing Assessment in the children from the SG and CG and *p*-value calculated for comparison according to the participants' ages (in years).

	Test	Group	N	Mean	Standard Deviation	Minimum	Maximum	Percentile 25	Median	Percentile 75	*p*-valor
		4	3	22,22%	19,24%	0,00%	33,33%	8,33%	33,33%	83,33%	
		5	5	60,00%	36,51%	0,00%	100,00%	66,66%	66,66%	100,00%	
	NVSM	6	11	72,72%	32,72%	0,00%	100,00%	66,66%	100,00%	100,00%	0,021*
		7	7	80,95%	26,23%	33,33%	100,00%	66,66%	100,00%	100,00%	
		8	5	100,00%	0,00%	100,00%	100,00%	100,00%	100,00%	100,00%	
		4	3	55,55%	50,92%	0,00%	100,00%	16,67%	83,33%	100,00%	
		5	5	53,33%	50,55%	0,00%	100,00%	0,00%	66,66%	100,00%	
	VSM	6	11	84,85%	17,41%	66,66%	100,00%	66,66%	100,00%	100,00%	0,498
SG		7	7	90,47%	16,27%	66,66%	100,00%	66,66%	100,00%	100,00%	
		8	5	80,00%	29,82%	33,33%	100,00%	66,66%	100,00%	100,00%	
		Tot	31	77,42%	31,49%	0,00%	100,00%	66,66%	100,00%	100,00%	
		4	3	86,67%	11,55%	80,00%	100,00%	80,00%	80,00%	95,00%	
		5	5	88,00%	17,89%	60,00%	100,00%	80,00%	100,00%	100,00%	
	SLT	6	11	92,73%	10,09%	80,00%	100,00%	80,00%	100,00%	100,00%	0,572
		7	7	94,29%	15,12%	60,00%	100,00%	100,00%	100,00%	100,00%	
		8	5	84,00%	16,73%	60,00%	100,00%	80,00%	100,00%	100,00%	
		Tot	31	90,32%	13,54%	60,00%	100,00%	80,00%	100,00%	100,00%	
		4	1	100,00%	0,00%	100,00%	100,00%	100,00%	100,00%	100,00%	
		5	2	83,33%	23,58%	67,00%	100,00%	66,66%	83,33%	100,00%	
CG	NVSM	6	3	100,00%	0,00%	100,00%	100,00%	100,00%	100,00%	100,00%	0,580
		7	3	88,89%	19,25%	67,00%	100,00%	66,66%	100,00%	100,00%	
		8	2	100,00%	0,00%	100,00%	100,00%	100,00%	100,00%	100,00%	
		Tot	11	93,94%	13,49%	67,00%	100,00%	100,00%	100,00%	100,00%	
		4	1	100,00%	0,00%	100,00%	100,00%	100,00%	100,00%	100,00%	
		5	2	83,33%	23,58%	67,00%	100,00%	66,66%	83,33%	100,00%	
	VSM	6	3	88,89%	19,25%	67,00%	100,00%	66,66%	100,00%	100,00%	0,836
		7	3	77,77%	19,25%	67,00%	100,00%	66,66%	66,66%	100,00%	
		8	2	83,33%	23,58%	67,00%	100,00%	66,66%	83,33%	100,00%	
		Tot	11	84,85%	17,41%	67,00%	100,00%	66,66%	100,00%	100,00%	
		4	1	80,00%	0,00%	80,00%	80,00%	80,00%	80,00%	80,00%	
		5	2	100,00%	0,00%	100,00%	100,00%	100,00%	100,00%	100,00%	
SLT	6	3	100,00%	0,00%	100,00%	100,00%	100,00%	100,00%	100,00%	0,040*	
	7	3	100,00%	0,00%	100,00%	100,00%	100,00%	100,00%	100,00%		
		8	2	100,00%	0,00%	100,00%	100,00%	100,00%	100,00%	100,00%	
		Tot	11	98,18%	6,03%	80,00%	100,00%	100,00%	100,00%	100,00%	

Legend: SG= study group; CG= control group; NVSS=non-verbal sound sequence; VSS= verbal sound sequence; SLT= sound localization test; Kruskal-Wallis test;

= statistically significant p-value fixed at 0.05

## DISCUSSION

The group of dysphonic children was made up based on the spontaneous demand from the Pediatric ENT Ward of the UNIFESP. The distribution and description of the children in this group showed predominance of boys and organic-functional dysphonia clinical picture (74.2%). As far as the Auditory Processing is concerned, 38.7% of this group had some change in the Simplified Assessment answers, while in the CG, 100% of the children showed normal results in this assessment (Table 1).

Upon the intergroup comparison (Table 2), we noticed the presence of a statistically significant difference between the groups in the NVSSM test, with a worse performance seen among dysphonic children. Deficits in the memorization of sound pattern sequences may be associated to the difficulty in distinguishing the sound relations of suprasegmental traces in the prosody speech, volume, intensity, tone^16^ and the difficulty in perceiving the differences between sound acoustic characteristics.

Therefore, similarly to other studies carried out with children who had learning disorders^8^, ^9^, ^11^, ^14^, ^15^, the results from this study may indicate that the voice changes are also associated with changes in auditory perception. Other studies carried out with dysphonic children showed similar results^2^, ^3^, ^4^.

The MSV assessment did not show statistically significant differences between the groups, as well as the SLT assessment. The good performance in the SLT assessment enables us to say that the group of dysphonic children had good possibilities of localizing the sound source and, by consequence, make an auditory discrimination of the sounds (Table 2).

The intragroup comparison and that of age ranges (Table 3) of the children in the different assessment tasks of the auditory function showed that in the SG there was an improvement in the NVSSM responses with the increase in age. These answers were also compared in two-by-two ages, by means of the Bonferroni's rule (Mann-Whitney test) seeing indicative values of trends of difference between ages 4 and 6; 4 and 7; 4 and 8; 5 and 8; 6 and 8. Thus, the children between 4 and 6 years of age had the worst performance in this test when compared to the older ones, which may indicate a delay in the maturation of the auditory pathways of this group of dysphonic children. The accuracy in the temporal ordering skill needs that both right and left hemispheres be anatomically and functionally intact. Moreover, this temporal processing mechanism is widely recognized among researchers as important in speech performance^12^.

The CG showed a statistically significant difference in the SLT test, when performance was compared between ages: the 4-year-old child did not properly identify the direction of the sound stimulus, scoring 80%; while all the other children scored 100% of correct answers. Notwithstanding, this statistical difference is not relevant; because 80% of correct answers is considered a normal result in the SLT test at this age.

Observing the mean value of the responses from each age range in the SG shows that the 4-year old child responded much below what was expected for children of that age according to literature parameters^16^; and in the comparison with the CG made up in this study. The same could be seen in relation to the 5-year-old dysphonic children. The progressive increase in the number of correct answers along the ages indicated that the auditory perception for instrumental sound sequence patterns improves with age, also in dysphonic children; and the literature also indicates regarding the development of normal children or those with other communication or learning disorders^7^, ^8^, ^9^, ^10^. This progressive increase in the number of correct answers may explain why the vocal rehabilitation difficulties of such small children improve with growth and development, since the increase in age may more easily help perceive the subtle characteristics of their own voice variations.

Based on the findings from this paper we recommend that in the speech and hearing assessment of dysphonic children, auditory processing must be included. Knowledge about the acoustic processing skill of sounds may help in the rehabilitation process, especially in strategies which stimulate the monitoring of the child's voice. From the therapeutic standpoint, the results from this study indicate that the association of vocal training strategies with the non-verbal temporal ordering auditory perception stimulation - associated with the distinction of sound relations of intensity, duration and frequency, among others - may facilitate the obtaining positive results, but efficient ones in working with dysphonic children.

## CONCLUSION

The auditory processing assessment of dysphonic children showed the presence of temporal ordering auditory skills of non-verbal sounds which differentiate this group from that of the comparison. The progressive increase in the number of correct answers with the increase with age showed that dysphonic children had the same auditory system maturation pattern that normal children or those with other communication disorders, which may explain the difficulties found in the vocal rehabilitation of younger children.
